# Examples of Weak, If Not Absent, Form-Function Relations in the Vertebrate Heart

**DOI:** 10.3390/jcdd5030046

**Published:** 2018-09-08

**Authors:** Bjarke Jensen, Theodoor H. Smit

**Affiliations:** Department of Medical Biology, Amsterdam Cardiovascular Sciences, University of Amsterdam, Amsterdam UMC, Meibergdreef 15, 1105AZ Amsterdam, The Netherlands; t.h.smit@amc.uva.nl

**Keywords:** evolution, development, physiology, structure

## Abstract

That form and function are related is a maxim of anatomy and physiology. Yet, form-function relations can be difficult to prove. Human subjects with excessive trabeculated myocardium in the left ventricle, for example, are diagnosed with non-compaction cardiomyopathy, but the extent of trabeculations may be without relation to ejection fraction. Rather than rejecting a relation between form and function, we may ask whether the salient function is assessed. Is there a relation to electrical propagation, mean arterial blood pressure, or propensity to form blood clots? In addition, how should the extent of trabeculated muscle be assessed? While reviewing literature on trabeculated muscle, we applied Tinbergen’s four types of causation—how does it work, why does it work, how is it made, and why did it evolve—to better parse what is meant by form and function. The paper is structured around cases that highlight advantages and pitfalls of applying Tinbergen’s questions. It further uses the evolution of lunglessness in amphibians to argue that lung reduction impacts on chamber septation and it considers the evolution of an arterial outflow in fishes to argue that reductions in energy consumption may drive structural changes with little consequences to function. Concerning trabeculations, we argue they relate to pumping function in the embryo in the few weeks before the onset of coronary circulation. In human fetal and postnatal stages, a spectrum of trabeculated-to-compact myocardium makes no difference to cardiac function and in this period, form and function may appear unrelated.

## 1. Introduction

Four weeks into human embryonic development, a single vessel connects the developing pulmonary vasculature to the left atrium [1]. Eleven weeks later, pulmonary venous tissue has been incorporated to the left atrium and four separate pulmonary veins now open to the left atrium [2]. This ontogenetic acquisition of veno-atrial connections varies between individuals and abnormal connections, for example three or five, occur in approximately one out of four people [3,4]. However, it is inconsequential to a person’s health whether there are three, four, or five pulmonary veins connecting to the left atrium [3], which suggests that there is no relation between the normal variation in number of pulmonary veins, i.e., the form, and its function. Streaming of blood in the left atrium will be impacted by the number of veins conveying blood to the lumen, but is this an important functional relation if there are no consequences to whole organ and body performance?

The example of the number of pulmonary veins illustrates some of the pitfalls of establishing relation between form and function. First, at what level of biological organization should form-function relations be assessed: on the level of the cell, tissue, chamber, organ, or organism? Second, which function should be assessed? Any structure of the body impacts on (parts of) the body and it is in principle possible to establish a consequence of the presence of the structure. However, consequences are different with regard to adaptation in evolutionary biology. An adaptation is a trait that has been favored by natural selection and, thus, relates directly or indirectly to the reproductive success of the organism [5]. Third, are we seeking proximal answers such as how the left atrial blood streams are affected by the number of pulmonary veins, or ultimate answers such as why (or better, how come) the number of pulmonary veins is variable?

When attempting to establish causality, Niko Tinbergen suggested the application of four types of questions, which are derivatives of the four categories of causes of Aristotle (given in parenthesis):

- Mechanism (material cause), how does it work?
  Example: The heart pumps blood.
- Function (finale cause), why does it work?
  Example: because it perfuses the tissues with blood.
- Ontogeny (formal cause), how is it made?
  Example: cardiogenic mesoderm surrounds a blood-filled cavity.
- Phylogeny (efficient cause), why did it evolve?
  Example: the propulsion of blood through the tissues compensates for the increased diffusion distance between tissue and environment associated with greater body sizes.

August Krogh proposed that for “a large number of problems, there will be some animal of choice or a few such animals on which it can be most conveniently studied” [6]. For instance, concerning the biology relevant to blood pressure, would one not want to study giraffes, which are the animals with the highest known systemic blood pressures [7]? In comparing different animal species, we may encounter evolutionary differences in mechanisms and functions that, if understood correctly, could make us understand the efficient cause of why something became successful. As always, it is important that a functional advantage is not assumed beforehand. For example, Gould and Lewontin [5] emphasizes that the question of “what did the *Tyrannosaurus rex* use its tiny front limbs for?” will likely receive an unsatisfactory and unfalsifiable answer. In contrast, we can reasonably answer “how did the *Tyrannosaurus rex* get its tiny front limbs” because the fossil record shows a conspicuous reduction of the front limbs concomitant with increments in the size of the hind limbs and head [5]. Viewed in this way, the reduction in the front limbs allows for the prioritization of (energy to) the hind limbs and head. Similarly, we are not inclined to ponder the use of the tiny limbs of ancestral snakes or remnants of the pelvic girdle among some families of extant snakes [8,9] because extant advanced snakes are successful without limbs. It is rarely considered for the heart, however, whether there are vestigial features without function [10].

Confusion and faulty reasoning, then, may arise from asking the wrong questions. Furthermore, ‘why’ questions can do us a disfavor by implying purpose. As stated in the quote attributed to Ernst Wilhelm van Brücke “teleology is the mistress that the biologist cannot live without but is too ashamed to be seen with in public.” Below, we will focus on trabeculated ventricular muscle because it exhibits more than one function, so we may ask which function is the salient one? It shows ontogenetic changes, so we may ask which life stage should we focus on when trying to establish form-function relationships. In addition, it shows phylogenetic changes, so we may ask what could be the cause of the reduction of trabeculated muscle that happened independently and together with the evolution of endothermy in mammals and birds.

## 2. Cases

### 2.1. One Form and Multiple Functions–Which Mechanism (Material Cause)?

#### The Curious Case of the Crocodylian Heart

**Figure 1:**
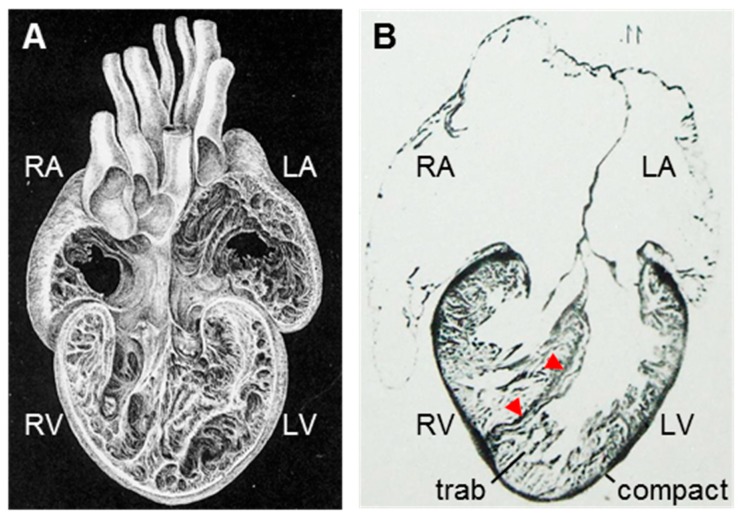

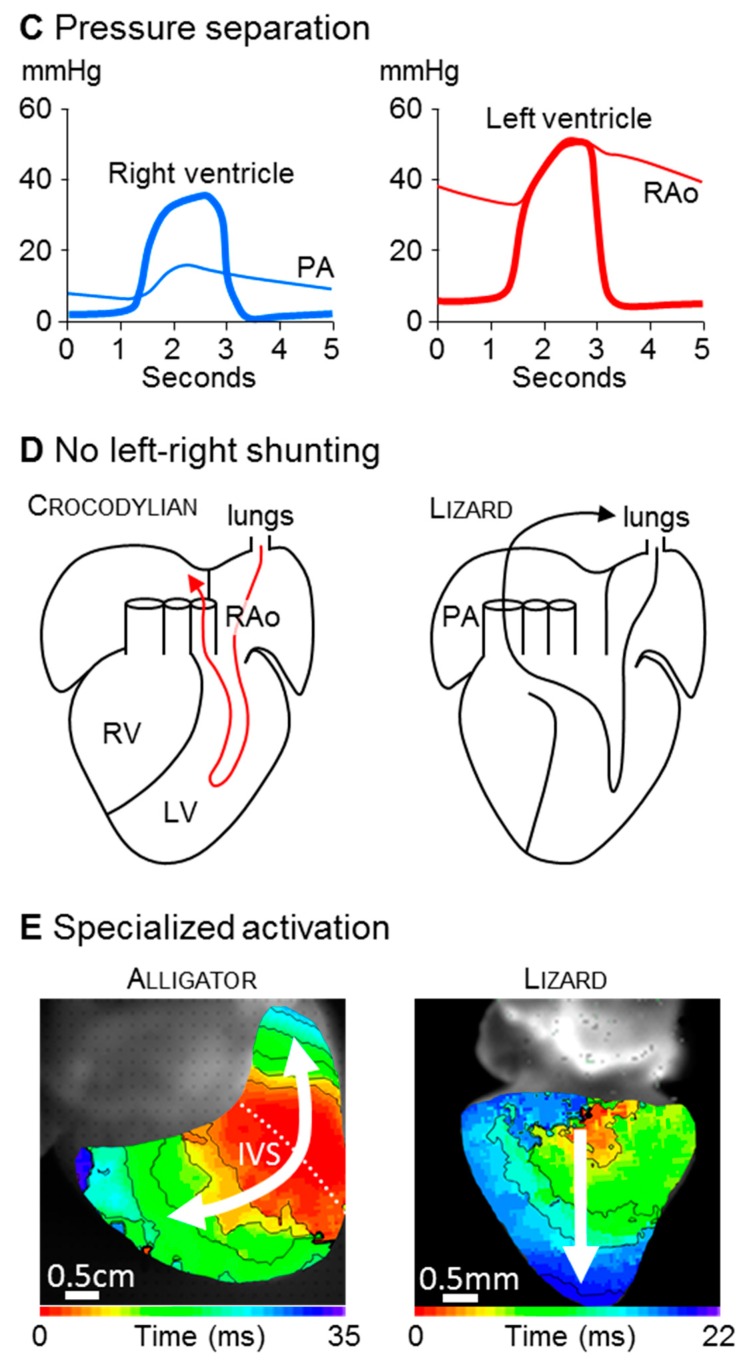
Consequences of the crocodylian ventricular septum at the level of the organ. (**A**) Both the left (LV) and right (RV) ventricle of crocodylians are extensively trabeculated. Adapted from [32], figure 10. (**B**) Histology shows the ventricular wall can be seen to consist of a thin outer shell of compact myocardium and an extensive layer of trabeculated myocardium (trab). The ventricular septum consists of a thin layer of tightly-organized myocardium (red arrowheads). Adapted from Reference [11], table IX, figure 11. (**C**) Blood pressures of the left and right ventricle and major arteries. Notice that the pulmonary artery (PA) has much lower pressures than the aorta (RAo). Redrawn from [16], figure 3b. (**D**) In crocodylians, blood returning from the lungs is always ejected through the right aorta (RAo) and left-to-right shunts are, therefore, impossible anatomically. In contrast, in non-crocodylian reptiles (here exemplified by a lizard), there is no full ventricular septum and the pulmonary venous blood can be shunted back to the lung circulation through the pulmonary artery. (**E**) Optical mapping of electrical activation (depolarization). The pattern of ventricular activation is specialized in the crocodylian ventricle compared to the setting of non-crocodylian reptiles (e.g., lizard) as it spreads laterally from the ventricular septum (IVS) rather than from the base to the apex. Adapted from References [14,23].

The crocodylian heart has a full ventricular septum, which distinguishes it from the hearts of all other ectothermic vertebrates (fishes, amphibians, and reptiles) that have been studied to date (Figure 1A,B) [11,12,13]. The ventricular septum has a membranous part and a larger myocardial part which, like all cardiac muscle, propagates the electrical impulse and contracts upon electrical activation [13,14]. Besides these two functions, the ventricular septum has at least three additional functional consequences at the organ level.

First, the blood pressure of the right ventricle can be substantially lower than that of the left ventricle (Figure 1C) [15,16,17]. This allows for low blood pressures in the pulmonary circulation, which, in turn, allows for a thinner blood-gas barrier at the respiratory epithelium [18].

Second, left-to-right shunting and the re-entry of pulmonary venous blood to the pulmonary circulation, which occurs in non-crocodylian reptiles, is anatomically impossible (Figure 1D) [12,13]. (Crocodylians often have right-to-left shunting where the right ventricle ejects a part or all of its stroke volume to the left aorta [19]). The absence of shunting improves the efficacy of oxygen transport [20,21].

Third, electrical activation spreads from the ventricular septum rather than from the base to the apex as in non-crocodylian reptiles (Figure 1E) [14,22,23].

Consequences of the ventricular septum can be established as above, but it is surprisingly difficult to ascertain its functional advantage besides the basic properties of myocardium (electrical propagation and contraction). The specialized manner of ventricular electrical activation does not shorten ventricular activation time as it does in mammals and birds [14]. Oxygen-consumption dependent behaviors are not limited by the level of shunting in crocodylians and reptiles [21,24,25]. The rates of oxygen consumption in crocodylians are not higher than in lizards despite the thin blood-gas barrier [26,27]. On the level of the organism, therefore, an advantage of the ventricular septum is not evident. It has then been proposed that the specializations of the crocodylian heart, including the ventricular septum, may have been selected for at a much earlier time in evolution when, presumably, crocodylians would have been much more active behaviorally [28]. Although this conjecture is difficult to test, it does emphasize the possibility that the conditions are passed in which the character provided an advantage to reproductive success.

The case of the crocodylian ventricular septum shows any one form may have multiple functional consequences. Ventricular septation in pythons also leads to pronounced pressure differences and a reduction of shunts [29,30]. In both cases, however, the functional advantage (final cause) on the level of the organism is not evident [31] and this calls into the question which form-function relation is the salient one.

### 2.2. Ontogeny (Formal Cause): When Is Form and Function Related?

We can postulate that, for any structure, there is one or several stages in ontogeny when the form-function relation is the strongest [33]. Such ontogenetic relations are implied in the term ‘immature,’ which means there may be early ontogenetic stages where a structure is not yet fully functional. In the chicken embryo, electrical propagation develops prior to cardiac contraction [34] and heart formation and pumping commence days before the circulation of plasma/blood is necessary for development, which is shown in embryos with an experimentally-ligated outflow tract [35]. The cessation of cardiac pumping by genetic perturbations in developing zebrafish and fruit flies have similarly shown that many early features of embryogenesis are not reliant on cardiac pumping [36,37] (although it has been proposed that pumping *per se* promotes proper heart morphogenesis [38], but this remains to be shown). At least in embryogenesis, then, structures may develop before they provide a functional advantage to the organism.

**Figure 2:**
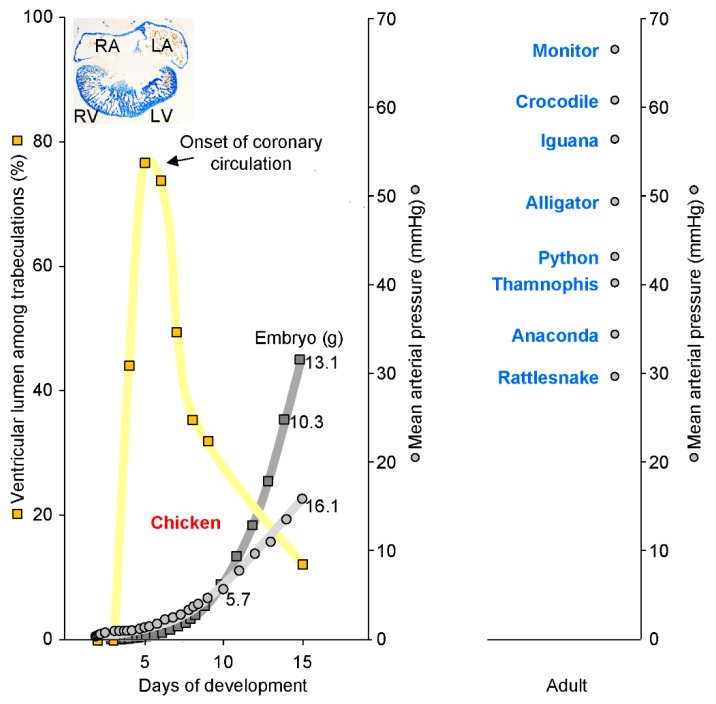
Highly trabeculated ventricles are capable of generating very substantial blood pressures. The left panel shows the *in ovo* development in chicken embryos of body mass (dark grey) and blood pressure (grey). In parallel, the wall architecture (yellow) changes from heart tube (2 to 3 days), to highly trabeculated (6 to 7 days), and to predominantly compact (15 days). The insert shows the highly trabeculated left (LV) and right ventricle (RV) at 6 days of development (and the almost a-trabecular left (LA) and right atrium (RA)). From approximately 6 days onwards, when blood pressure is about 3 mmHg, trabeculations and blood pressure become inversely related. The right-hand panel, however, shows that ectotherms have much greater blood pressures than developing chicken despite having highly trabeculated ventricles. Assuming that the cardiac mass is a fixed proportion of embryonic weight, which it is in a human [57], blood pressure will have a tight relation to cardiac mass (R^2^ = 0.96, blood pressure and embryo weight). In the left panel, values for body mass and blood pressure are from Reference [58] and values for ventricular wall architecture are from Reference [45]. In the right panel, the blood pressures of python are from Reference [59] and the remaining values from Reference [60]).

We can also envision the inverse scenario where structures provide a functional advantage in early stages of ontogeny but they are inconsequential to the adult animal. This could be the case for the caval vein myocardium [10] and the extent of trabeculated to compact myocardium of the ventricles of mammals may be another case. In the embryo, the ventricle is without coronary circulation. To maintain homeostasis, any myocardium must, therefore, be in close proximity to the blood of the ventricular lumen. Trabeculated myocardium is then a solution to growth without a coronary circulation because it is bathed in the blood returning to the heart (Figure 2) [39]. In the embryo, as shown in mice, the trabeculated myocardium is richer in mitochondria than the compact wall, which suggests that it contributes the most to ventricular function [40]. Unsurprisingly, inhibition of trabecular growth and, therefore, ventricular growth causes hypoplastic ventricles that associate with gestational retardation and lethality [41]. However, in slightly older stages of development, the coronary circulation is established and the trabeculated myocardium will have much reduced proliferation. Subsequent growth of the ventricle occurs almost exclusively by the compact wall (Figure 2) [42,43,44]. Some trabeculated myocardium persists in the adult heart, but it is proportionally much reduced [45]. If cardiac functional measures such as mean arterial pressure are assessed during gestation, their development is tied to the size of the heart, but, once coronary circulation is established, they are not tied to the proportion of trabeculated myocardium (Figure 2). The ontogeny of trabeculated myocardium in endotherms suggests, first, that trabeculated myocardium is tremendously important in an early and brief period only and, second, that it persists once formed. In the adult heart, trabeculations have a limited ability to remodel [46] and likely contributes to ventricular function on par with the compact wall [47].

It is not fully clear how embryonic trabeculations exactly relate to the trabeculated muscle of the adult heart [48]. Lineage studies in mice have shown that the papillary muscles and the Purkinje cells of the peripheral ventricular conduction system are derived from embryonic trabeculations [49,50]. Non-papillary and non-Purkinje trabeculated muscle of the adult ventricle is also derived from embryonic trabeculations, but some of the trabeculated muscle may have a substantial capacity to give rise to compact wall myocardium [51]. Conversely, when trabeculations start to form, they are derived from the heart tube, which expresses genes such as *Hey2* that are also expressed later in the compact wall [44,52,53]. The trabecular and compact layers, therefore, share origin in the early heart tube and, in later stages of development, they may have some capacity to intermingle even though the compact wall was not much affected after an experimentally-induced reduction of proliferation in the formed trabeculated myocardium [54]. Much is being learned from lineage studies in mice notwithstanding that some insights are difficult to reconcile possibly because the tracings are tied to different genes, they are induced at different developmental ages, and mice strains differ in the extent of trabeculated muscle [55]. Nonetheless, the left ventricles of mice and human differ in the proportion of trabeculated to compact muscle [45] and the importance of compaction, i.e., capacity of trabeculated muscle to give rise to a compact wall, remains a controversial topic [47,56].

### 2.3. Form Unrelated to Function: No Final Cause?

#### Trabeculation of the Left Ventricle of Adult Humans

**Figure 3:**
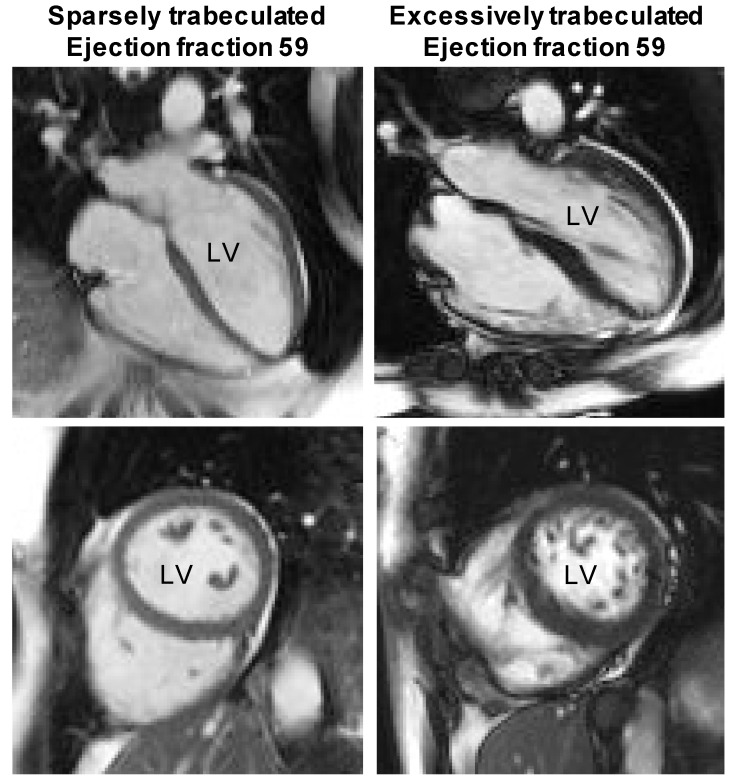
The architecture of the human left ventricle (LV) appears to have no impact on function. Normal ejection fractions (stroke volume/end-diastolic volume) occur in ‘Sparsely trabeculated’ and ‘Excessively trabeculated’ ventricles alike. The top row shows so-called four-chamber views and the bottom row shows transverse views of the two ventricles. Note the much more numerous and extensive trabeculations in the images on the right. Images from published data sets [45].

The left ventricle of adult humans is composed of a compact wall with a network of trabecular myocardium on its luminal side (Figure 3). Most prominent of the trabecular myocardium are the papillary muscles, which anchor the atrioventricular valve leaflets [61]. The non-papillary trabecular network can vary in extent from very meager to extensive and may make up between near-zero percent to some 25% of the left ventricular mass (Figure 3) [62]. Ventricular wall architecture is commonly measured as the ratio of the transmural thickness of the trabecular-to-compact wall. Analysis of thousands of cohort participants have shown that the ratio has a log-normal distribution in the population [47]. Surprisingly, the ratio is not related to functional measures like ejection fraction and blood pressure [63,64] or is so poorly related that it is deemed clinically irrelevant [47]. Even in cases of excessive trabeculation, which can be diagnosed as left ventricular non-compaction cardiomyopathy [65,66], there is little or no relation between the extent of trabecular myocardium and function [62,64]. Furthermore, adverse outcomes like sudden cardiac death also appear to not be related to the extent of trabecular myocardium but instead to ventricular dilation and fibrosis [47,63,67]. In adult humans, then, the left ventricular wall architecture is likely not related to function on the level of the organ and the individual when it is measured as proportions of trabecular and compact muscle [68]. This conjecture is supported by the meta-analytical finding that systemic blood pressure is similar across mammal phylogeny and body size [69] despite a substantial variation in the extent of trabeculation [70].

Already Pettigrew noted that the compact ventricular muscle of mammals and birds appears to be wound around the cavity of the left ventricle [71]. This tissue organization was described by Streeter as geodesic lines derived from the theorem of Clairaut [72] and such organization is still thought to be in a close relationship with pumping function [73]. However, Streeter recognized the difficulty of extending his analysis to the “free trabeculata” because the Clairaut constants became “irregular” [72]. The “irregular” values could be taken to mean that the trabeculated muscle contributes less to pumping function than the compact muscle. However, it could also mean that the architecture of the trabeculated muscle is poorly analyzed as extensions of geodesics that wind around the entire left ventricular cavity [74,75]. Instead, the trabeculations may surround the inter-trabecular spaces in approximate geodesic lines locally rather than globally. Such relations have not been analyzed to the best of our knowledge, but the trabeculated muscle of ectotherms does appear as intertwined arches and it is certainly functional despite that its organization differs from that of the compact wall [60,76,77].

### 2.4. Relationship between Form and Function in Evolution (Efficient Cause)?

#### Ventricular Trabeculations in Vertebrates

The inverse relation of the trabeculated wall architecture and blood pressure between 6 and 15 days of development in the chicken embryo is shown in Figure 2 and suggests that coronary circulation favors compact wall growth and causes a decrease in the proportion of trabeculated myocardium. This could be the case in the ontogeny of mammals and birds. Coronary vasculature is found in most ectotherms and can be found within the trabeculated myocardium of the extensively trabeculated ventricles [78,79,80]. This comparative analysis suggests that coronary vascularization is a necessary condition for the prioritization of the compact architecture over the trabeculated architecture, but it is not a sufficient condition. In fact, the adaptive value of compact architecture over the trabeculated architecture is not clear, i.e., the functional advantage (final cause) and explanation for its evolution (efficient cause).

**Figure 4:**
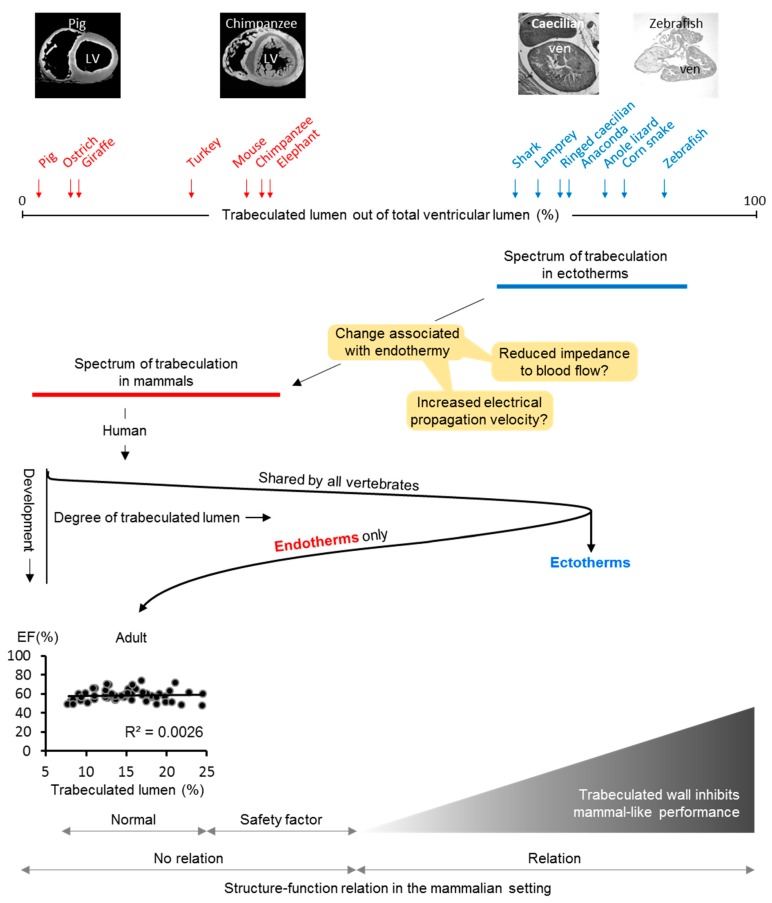
Evolutionary transition from trabeculated to compact chamber walls. In the evolution from ectothermy to endothermy, the cardiac chambers became much less trabeculated and the chamber walls became compact. This change (orange boxes) may have been impacted on the heart by reducing impedance to flow and accelerated chamber activation times, both of which would facilitate a short cardiac cycle and, thus, the high heart rates that characterizes the endotherms. In development, all vertebrates form highly trabeculated chambers, but, only in endotherms, is this design reversed by a temporary slow-down in proliferation of the trabeculated muscle while proliferation is maintained in the compact wall [42,43,44]. In the adult (human), there is variation in the extent of trabeculated muscle, but this extent does not associate with functional measures. Nonetheless, extremely excessive trabeculated ventricles, ectotherm-like, appear incompatible with life in endotherms [88]. This suggests that the extent of trabeculated myocardium in endotherms will have a normal range, a safety factor range where excessive extent of trabeculated myocardium is not pathological, and an ectotherm-like range, which will be detrimental to pumping function, possibly because of the impairment of filling and emptying. This is partly based on and adapted from Reference [45].

As trabeculated as a human left ventricle with excessive trabeculations can be (Figure 3), its walls are much less spongy than the ventricular walls of ectotherms (Figure 4) [45]. In ectotherms, the individual trabeculations are typically less than 50 µm wide as is the space between trabeculations [45]. Such structure-cavity relations can be compared to trabecular bone and the size of grains in very fine sand and the distance between them [81]. Conversely, in endotherms, the trabeculated muscle can be orders of magnitude greater and pebble-like rather than sand-like [45,82]. Most of the blood in the ventricles of ectotherms is between trabeculations, and, in endotherms in contrast, most of the blood is in the central lumen, which is without trabeculations [45]. There are at least two primary functions attributable to trabeculated myocardium: propagation of the electrical impulse and contraction [23,83]. The extensively trabeculated ventricles of some species of ectotherms generate systolic blood pressures approaching those of mammals (Figure 2) [84,85] and even the tubular hearts of earthworms can generate blood pressures that exceed 70 mmHg [86]. It is not clear, therefore, that the evolution of compact walls directly facilitated the generation of high ventricular blood pressures. Instead, the formation of compact walls yields a trabeculations-depleted ventricular cavity. Such a cavity (which holds a few ‘pebbles’) will offer less impedance to blood flow than the ‘sand-filled’ ventricle of ectotherms. We have, therefore, proposed that the architectural change from trabeculated to compact wall has allowed for a faster filling and emptying of the ventricles and, thus, it has facilitated the high heart rates that characterize endotherms (Figure 4) [87]. Higher heart rates allow for greater cardiac outputs and, therefore, higher blood pressure, which links the compact wall architecture to blood pressure via heart rates. Additionally, the faster chamber activation of endotherms is not explained by higher body temperatures and conduction systems only, which suggests a role for compact walls (Figure 4) [82].

### 2.5. Can A Reduction in Energy Expenditure Have A Structural Consequence?

#### The Outflow Tract of the Fish Heart

**Figure 5:**
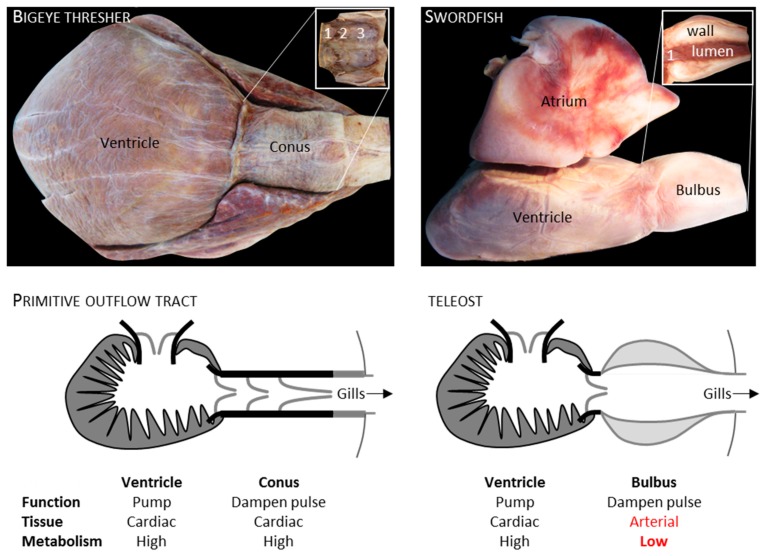
Arterial pole of fish hearts. The heart (158 g) of a 304 cm long bigeye thresher (*Alopias superciliosus*), a shark, had a myocardial outflow tract (Conus, ventral view) that comprised 8.9% of the cardiac mass. In the insert, the conus has been isolated, cut longitudinally, and folded out, exposing its three rows of valve leaflets (1–3, 1st to 3rd row of valve leaflets). The heart of an approximately 50 kg swordfish (*Xiphias gladius*), a teleost, had an arterial outflow tract (Bulbus, view from the right). In the insert, the interior is exposed of the left half of the bulbus showing its thick arterial wall (1, only 1 row of valve leaflets). The categorization of metabolism as being high or low is based on values for mammals [96,97].

In the evolution of teleost fish, there was a change to the arterial pole whereby the myocardial outflow tract called the conus arteriosus disappeared and a pear-shaped arterial outlet called the bulbus arteriosus took its place (Figure 5) [89]. It is debated whether the bulbus of teleost fish should be considered a modification of an older structure or an evolutionary novelty, but the bulbus is evidently much more developed in teleosts than in non-teleosts [90,91]. A recent study shows in embryonic teleost fish that the myocardial outflow tract undergoes reprogramming to an arterial phenotype mediated by the gene *elnb* [92]. Despite the advances in understanding the phylogenetic appearance, the ontogenetic change, and the mechanism behind it, the functional advantage of an arterial bulbus over a myocardial conus remains elusive. Both the bulbus and conus work as a pressure and flow reservoir that ensures blood flow in the diastolic interval between ventricular ejections [93,94].

A key difference may be that sarcomeric contraction is the basis of conus function while recoil of elastic elements is the basis of bulbus function [93,94]. Given that the conus comprises between 10% to 30% of the cardiac mass [95] and the metabolic rate of heart muscle [96] is an order of magnitude greater than of arterial wall [97] (values for mammals), having the myocardial conus may be energetically more demanding than having the arterial bulbus (Figure 5). If the bulbus is stiff, ventricular ejection could become expensive, but it is highly compliant and can accommodate a large fraction of cardiac output [98]. To make a theoretical calculation of the energy saved by having a bulbus, we can assume that the disappeared conus was relatively small (10% of cardiac mass), that the metabolic rate of the bulbus was a tenth of the conus, and that the bulbus is relatively noncompliant and more expensive to fill than a conus. The change from conus to bulbus may then constitute a 5% reduction in stroke work, which is the energy spend by the ventricle on ejection in one cardiac cycle. Stroke work in ectotherms is approximately 8 mJ per kg body mass, heart rate of a 3 kg sockeye salmon is approximately 40 beats per minute [99,100], and the saved energy in a course of a year then amounts to approximately 25.000 J or multiple catches of zooplankton, which is a mainstay of the food of sockeye salmon [101]. These considerations allow for the conjecture that the adaptive value of the bulbus is a low energy demand. If so, the form and function of the bulbus are then secondary consequences.

### 2.6. Lungs, Then Heart

#### Can Loss of the Lungs Impact on Cardiac Septation?

Loss of lungs evolved among amphibians in some aquatic caecilians and salamanders, presumably to reduce buoyancy in fast-flowing water where oxygen is abundant [102,103]. Besides having no lungs, these lungless amphibians also exhibit reduced septation in the atria and the outflow tract [102,104]. We can then ask whether the reduction in cardiac septation is an adaptation to loss of lungs or whether the reduction of the lungs causes a reduction in cardiac septation. In all lunged vertebrates, the pulmonary vein develops in the dorsal mesocardium, which is a bridge of mesenchyme between the developing atria and the pharyngeal mesoderm in which the lungs develop [105]. The dorsal mesocardium projects into the atrial lumen as the dorsal mesenchymal protrusion, which is necessary for the closure of the primary foramen between the left and right atrial cavity [106,107]. Accordingly, a poorly developed dorsal mesenchymal protrusion leads to atrial septal defects [108]. Furthermore, the pharyngeal mesoderm also contributes cells to the arterial pole of the heart [109]. These observations allow for the conjecture that the reduced cardiac septation of lungless amphibians is a direct consequence of the reduced development of the pharyngeal mesoderm [104] rather than an adaptation to intracardiac flow patterns in the absence of a pulmonary circulation.

## 3. Synthesis

We have attempted to apply the four causes of Aristotle that Tinbergen adapted to biology. Our motivation came from the confusion and conflicting views concerning the role of trabeculated muscle in vertebrate hearts. ‘Function’ is a concept that often implies purpose and teleology may exacerbate the confusion. The application of the four causes has revealed that function can be interpreted in several ways. In part, this is because function may be assessed under conditions that are not conducive to test a particular form-function relation. While our cases are based mostly on evolutionary biology, we would argue that there is relevance for medicine. Left ventricular non-compaction cardiomyopathy is characterized as a distinct form of cardiomyopathy by the American Heart Association and is diagnosed when trabeculated myocardium is excessive [65,66]. (Non-compaction cardiomyopathy has also been reported for a domestic cat [110]). Yet, in patients and healthy subjects, left ventricular function does not correlate with the extent of trabeculated myocardium and the diagnostic criteria have very poor sensitivity [63,64,67,111,112]. These observations suggest that form and function are not related in the human heart when we consider the extent of trabeculated myocardium. However, compact and trabeculated muscle is likely to be equally functional and, as argued above, when we consider all life stages, a credible argument can be made that function is tightly related to the extent of trabeculated muscle in the embryonic heart before coronary circulation is established. Therefore, we propose that trabeculated muscle is functional in all life stages, but the extent of trabeculated muscle relative to the compact wall is related to function in certain life stages only. If this conjecture is true, it may be futile to use and develop morphometrics to identify true cases of left ventricular non-compaction cardiomyopathy. Functional assessment remains the crucial readout.

Using the ventricular septum of the crocodylians as a case, we argued that any one feature may have more than one function. One of the functions is the recently described specialized manner of electrical propagation. The crocodylian septum then exemplifies a case where we may first ask whether there could be salient functions that have not been characterized? Second, which of the known functions should be assessed in order to identify the adaptive value of a structure or should all functions be assessed? In this context, it is interesting to note that considerable effort has been spent on showing the adaptive value of right-to-left shunting in crocodylians without a clear-cut adaptive value being shown [21].

In most instances, there are grounds for assuming that a structural change to the heart relates to a functional change. However, as argued for the case of loss of lungs in amphibians, it is entirely conceivable that a primary adaptation, lunglessness, induces a reduction of cardiac septation (in the atria and the outflow tract) because of developmental changes to the mesoderm that gives rise to both lungs and cells of the cardiac septa. That is, if cardiac changes are seen in isolation, we may miss the primary adaptation. It also follows that it may be futile to assess the functional advantage of the reduction of the cardiac septa because the advantage may not be there anymore. Similar concerns for establishing form-function relations can be expressed when trying to understand the significance of the myocardial to arterial identity change of the outflow tract of fishes. Possibly, the primary adaptation relates to energy consumption with form-function relations being secondary.

In conclusion, we propose a negative derivative of the Krogh principle [6] namely, that for a form-function relation, there will be a large number of animals of choice and life stages on which it cannot be studied.
